# Research on Microscale Vehicle Logo Detection Based on Real-Time DEtection TRansformer (RT-DETR)

**DOI:** 10.3390/s24216987

**Published:** 2024-10-30

**Authors:** Meiting Jin, Junxing Zhang

**Affiliations:** 1 College of Information and Communication Engineering, Dalian Minzu University, Dalian 116600, China; 202311051003@stu.dlnu.edu.cn; 2 College of Computer Science and Engineering, Dalian Minzu University, Dalian 116600, China

**Keywords:** vehicle logo detection, RT-DETR, microscale datasets

## Abstract

Vehicle logo detection (VLD) is a critical component of intelligent transportation systems (ITS), particularly for vehicle identification and management in dynamic traffic environments. However, traditional object detection methods are often constrained by image resolution, with vehicle logos in existing datasets typically measuring 32 × 32 pixels. In real-world scenarios, the actual pixel size of vehicle logos is significantly smaller, making it challenging to achieve precise recognition in complex environments. To address this issue, we propose a microscale vehicle logo dataset (VLD-Micro) that improves the detection of distant vehicle logos. Building upon the RT-DETR algorithm, we propose a lightweight vehicle logo detection algorithm for long-range vehicle logos. Our approach enhances both the backbone and the neck network. The backbone employs ResNet-34, combined with Squeeze-and-Excitation Networks (SENetV2) and Context Guided (CG) Blocks, to improve shallow feature extraction and global information capture. The neck network employs a Slim-Neck architecture, incorporating an ADown module to replace traditional downsampling convolutions. Experimental results on the VLD-Micro dataset show that, compared to the original model, our approach reduces the number of parameters by approximately 37.6%, increases the average accuracy (mAP@50:95) by 1.5%, and decreases FLOPS by 36.7%. Our lightweight network significantly improves real-time detection performance while maintaining high accuracy in vehicle logo detection.

## 1. Introduction

Vehicle recognition plays a vital role in ITS, enabling comprehensive detection, management, and optimization of urban traffic by accurately identifying and analyzing vehicle characteristics such as license plates, models, logos, and colors. Although license plate recognition has been widely and successfully implemented, its effectiveness can be compromised by factors such as removal, obscuration, or tampering. In contrast, vehicle logos have emerged as a significant focus in vehicle recognition research owing to their uniqueness and stability. For small-scale objects, vehicle logo detection holds a critical position within the broader field of small-object detection.

In real-world scenarios, vehicle logos constitute only a small portion of an overall image. Traditional object detection algorithms often struggle with this, requiring a higher image resolution for accurate detection. Consequently, many existing vehicle-logo datasets contain objects larger than 32 × 32 pixels. For example, the HFUT-VL [1] dataset features logos sized at 64 × 96 pixels, the XMU [2] dataset includes logos sized at 70 × 70 pixels, and the VLD-45 [3] dataset has logos of approximately 40 × 32 pixels. Although these datasets perform well in close-range scenarios, vehicle logos in practical applications are often smaller and subject to occlusion and angle variations, making accurate detection challenging. Thus, building a dataset for long-range vehicle logo detection is crucial for advancing ITS development.

Early research relied primarily on manual feature extraction techniques, such as the Histogram of Oriented Gradient (HOG) [4], invariant moments [5], and Scale-Invariant Feature Transform (SIFT) [6]. However, these methods often require specialized detectors tailored to specific vehicle logo characteristics. As a result, these methods are limited by poor generalizability, a lack of robustness, and the inability to learn autonomously. To address these issues, deep learning methods have become prevalent, showing high efficiency in object-detection tasks [7]. CNNs have been widely optimized in terms of network depth and structure, resulting in the development of powerful models, such as ResNet [8], MobileNet [9], and ShuffleNet [10]. Detectors based on CNN architectures have evolved from two-stage models [11,12] to single-stage models [13,14,15,16,17]. All of these detection algorithms have made significant progress in both speed and accuracy, but their effectiveness in small-object detection still needs to be improved.

CNN-based vehicle logo recognition studies often struggle to capture global information effectively, whereas Transformers utilize self-attention mechanisms and parallel computations to enable global feature representation [18]. Originally developed for Natural Language Processing, the Transformer architecture was later adapted for Computer Vision with the introduction of the Vision Transformer (ViT), offering a new approach to feature extraction and advancing multimodal domain research [19]. The Transformer-based end-to-end object detector DETR improves model efficiency and performance by learning object localization and classification through a self-attention mechanism, eliminating the need for manually designed anchors and non-maximum suppression (NMS) components in the traditional detection pipelines [20]. Researchers subsequently developed various DETR variants to further optimize the model, by employing techniques such as unsupervised pretraining [21] and sparse sampling [22]. One notable advancement is RT-DETR, a real-time end-to-end object detector that leverages an efficient hybrid encoder with cross-scale fusion, significantly improving speed and outperforming the YOLOv8 detector in terms of accuracy.

Despite improvements in accuracy and speed, challenges persist in long-range small object detection, particularly with low detection accuracy and real-time performance owing to the high computational cost of encoder layers and self-attention mechanisms. To address these challenges, we propose an improved method based on RT-DETR. The main contributions of this study are as follows:

- The backbone network follows a “deep and thin” principle, leveraging ResNet-34 in conjunction with the lightweight CGBlock and the aggregated multilayer perceptron SENetV2. This combination enhances both shallow feature extraction and global feature representation, helping to preserve small object details through the integration of semantic and spatial information.
- The neck network adopts a Slim-Neck structure that incorporates the ADown block as a substitute for traditional downsampling convolutions. This modification streamlines the network architecture while preserving semantic consistency.
- A novel microscale vehicle logo dataset (VLD-Micro) featuring vehicle logos that are significantly smaller than those in traditional datasets, with an average size of 24 × 19 pixels, was introduced.
- Experiments conducted on the VLD-Micro dataset demonstrated that our model achieved a 1.6% higher mAP@50:95 than YOLOv8 and 7.4% higher than Faster R-CNN, with significantly fewer parameters. Relative to the original model, the mAP@50:95 increased by 1.5%, while the parameter count was reduced by approximately 37.6%, and the FLOPS decreased by 36.7%.

## 2. Related Works

### 2.1. Vehicle Logo Detection

Vehicle logo detection methods can be categorized into traditional and deep learning-based methods.

Traditional methods for vehicle logo detection usually rely on hand-designed features and utilize classical machine learning algorithms. For example, Psyllos et al. [23] proposed a method based on the SIFT operator that detects logos through feature matching and hypothesis testing. However, this approach, while clear in terms of feature representation, is computationally intensive. Peng et al. [24] utilized Statistical Random Sparse Distribution (SRSD) features combined with multiscale scanning for logo detection. Satzoda et al. [25] utilized the symmetry of a vehicle’s vertical midline to detect logos by identifying regions of interest (ROIs) in highly symmetric images. Yuan et al. [26] employed HOG + SVM algorithms to achieve effective detection. Yu et al. [1] developed a method that integrates OEM and LBP features for vehicle logo detection. Maha et al. [27] extracted vehicle logos via k-medoid clustering, removed background and noise, applied the wavelet transform and DCT to derive features from the transformed binary images, and finally matched these features via the Canberra distance. However, these methods are often sensitive to changes in illumination and viewing angles, which hinders their ability to capture the complex textures and shapes of vehicle logos effectively, ultimately leading to poor robustness and accuracy compared with traditional methods.

With the development of deep learning, object detection methods based on this technology have increasingly supplanted traditional methods. Compared with traditional techniques, these methods can autonomously learn features and offer superior robustness and generalizability, making them more suitable for real-time vehicle logo detection. Deep learning-based vehicle logo detection algorithms are primarily categorized into CNN-based methods and Transformer-based methods. CNN-based detection algorithms can be divided into two-stage and single-stage approaches. Two-stage algorithms, such as R-CNN [28], Fast R-CNN [12], and Faster R-CNN [29], first generate multiple ROIs within an image and then verify these regions through feature extraction to determine the object’s location and class. Huang et al. [2] utilized a Faster R-CNN for the recognition of vehicle logos. Single-stage algorithms, led by YOLO, integrate localization and classification into a single network to achieve real-time object detection [30]. Although the accuracy of single-stage algorithms was initially lower, ongoing research and improvements have brought their accuracy to levels comparable to or even exceeding those of two-stage algorithms. Chen et al. [31] proposed a joint framework based on capsule networks to increase accuracy in complex scenarios. Zhou et al. [32] introduced an effective method for detecting vehicle logos in motion-blurred environments by combining filter-DeblurGAN and VL-YOLO. By improving YOLOv4 and introducing a CSPDenseNet backbone network and deformable convolution, Jiang et al. [33] improved the detection accuracy by 5.72%. Song et al. [34] developed a VLD method based on the YOLO-T model and vehicle spatial structure correlation. Mainstream Transformer-based object detection algorithms, such as DETR, discard complex postprocessing such as NMS to achieve true end-to-end detection. Li et al. [35] achieved high accuracy using Swin Transformer, benefiting from its efficient computational and global feature-modeling capabilities. The Transformer framework captures global information that cannot be obtained by the CNN framework through parallel computation and a unique attention mechanism, offering a true end-to-end detection process. Therefore, for vehicle logo detection, the DETR algorithm provides more efficient real-time detection performance.

### 2.2. RT-DETR

DETR transforms the object detection task into a sequence-to-sequence problem by leveraging the Transformer architecture to directly predict object locations and classes from global image information. Compared with traditional two-stage object detection methods, DETR omits the need for candidate region extraction and feature matching. Compared with single-stage algorithms, DETR excels in capturing contextual relationships between objects and avoids complex operations such as anchor boxes and NMS. However, DETR’s attention mechanism involves a large number of parameters, which can lead to slower training convergence and reduced effectiveness in detecting small objects.

To address these challenges, researchers have proposed various improvements to optimize the DETR algorithm. Deformable DETR [36] combines deformable convolution and multiscale feature pyramids by replacing the attention module in DETR with a multiscale deformable attention module. This approach significantly reduces the computational complexity but increases the number of encoder tokens by a factor of 20. Efficient-DETR [37] reduces the number of decoder layers by employing dense and sparse set-based networks, although it results in a twofold increase in the number of GFLOPs compared with the original DETR. SMCA-DETR [38] employs a regression-aware mechanism to improve the convergence speed, whereas WB-DETR [39] employs a backboneless pure Transformer detector. Despite these significant improvements in detection speed, both methods still face challenges in terms of convergence speed and small object detection. CF-DETR [40] aims to improve the localization accuracy of small objects by refining coarse features. However, the addition of three new modules can reduce the overall detection efficiency.

**Figure 1:**
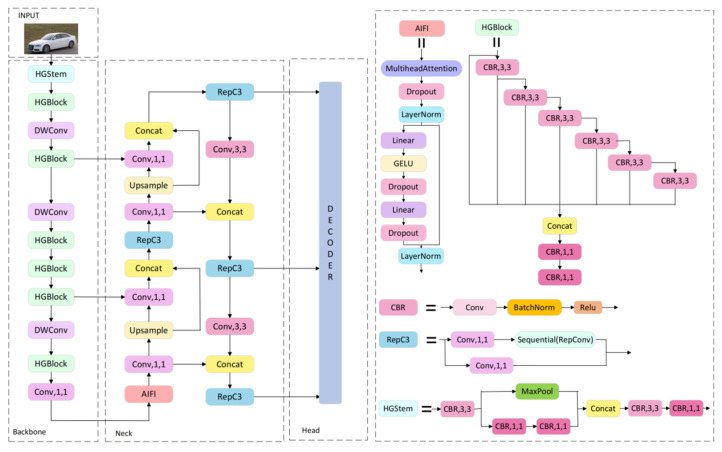
Structure of RT-DETR. The backbone comprises HGStem and HGBlock. The neck refines the features using CCFM and AIFI modules. The model includes three detection heads.

Despite ongoing advancements in object detection algorithms, achieving high detection accuracy for small objects often comes at the cost of reduced detection speed. RT-DETR addresses this issue by integrating the key components of object detection to maintain high accuracy for small objects while also achieving high detection speeds. Figure 1 shows the structure of the RT-DETR. The backbone network of RT-DETR produces three feature maps of different sizes with downsampling factors of 8, 16, and 32 relative to the input image resolution. The neck network includes a Transformer encoder layer that features two main components: the AIFI and the CCFM. AIFI reduces computational redundancy by performing intrascale interactions solely on the $S_{5}$ feature map. Specifically, the 2D feature $S_{5}$ is reshaped into a 1D vector, processed through a multihead attention mechanism and a feed-forward network (FFN), and then reshaped back into a 2D feature denoted as $\;F_{5}$. This feature is subsequently processed by the CCFM for cross-scalar feature fusion. The CCFM, similar to FFN and PAN structures, consists of two 1 × 1 convolutions and N RepBlocks. The depth and width of the RepBlocks as well as the encoding dimension of the encoder can be adjusted by varying the number of RepBlocks and encoding dimensions of the encoder. Adjustments to the backbone network facilitate detector scaling. The fusion block consists of N RepBlocks with outputs from the two paths combined via elementwise addition. This process can be formulated as follows:

(1) $$Q=K=V=Flatten(S_{5})$$

(2) $$F_{5}=Reshape(Attn(Q,K,V))$$

(3) $$Output=CCFM(\{S_{3},S_{4},F_{5}\})$$

where Q, K, and V refer to the query, key, and value matrices, respectively, all of which are derived from the flattened $S_{5}$ feature map. Attn represents multihead self-attention, whereas Reshape represents the process of restoring the feature map to its original shape, effectively reversing the flattening operation applied to $S_{5}$.

IoU-aware query selection improves detector performance by guiding the model during retraining to assign higher classification scores to features with high IoUs and lower scores to those with low IoUs. The optimization objective is as follows:

(4) $$L(\hat{y},y)=L_{box}(\hat{b},b)+L_{cls}(\hat{c},\hat{b},y,b)=L_{box}(\hat{b},b)+L_{cls}(\hat{c},c,IoU)$$

where $\hat{y}\;$ and y  denote the predicted and ground truths, respectively, and $\hat{y}=\{\hat{c},\;\hat{b}\}\;$ and $y=\left\{c,\;b\right\},\;c\;$ and b represent the categories and bounding boxes, respectively. IoU scores were introduced into the objective function of the classification branch to enforce consistency constraints between the classification and localization of positive samples.

## 3. Method

### 3.1. RT-DETR Improvements

To select the appropriate backbone, we conducted preliminary experiments under the same conditions as in Section 4, using the VLD-vicro dataset. As shown in Table 1, ResNet-34 achieved an mAP@50 of 0.966, an mAP@50:95 of 0.688, and a high FPS of 66.11, outperforming RT-DETR-L, ResNet-18, and ResNet-50. Therefore, ResNet-34 was chosen as the final model for further analysis in Section 4.

**Figure 2:**
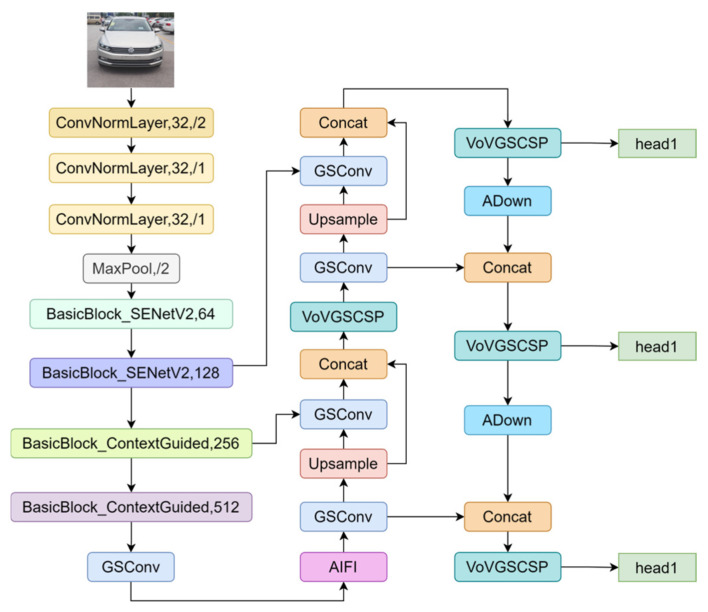
Improved structure of RT-DETR. The backbone includes ConvNorm, SENetV2, and Context Guided Blocks. Neck processes features using GSConv and AIFI, with VoVGSCSP and ADown for further refinement.

In this study, we developed a lightweight and efficient network for vehicle logo detection in long-range scenarios by enhancing both the backbone and neck layers of RT-DETR. The enhanced RT-DETR network is shown in Figure 2.

The backbone network was enhanced with the aggregated multilayer perceptron SENetV2 module and a Context Guided (CG) Block. Additionally, the neck network employs a lightweight Slim-Neck architecture in combination with the ADown block, resulting in a more streamlined overall design.

### 3.2. Backbone Improvements

#### 3.2.1. SENetV2

**Figure 3:**
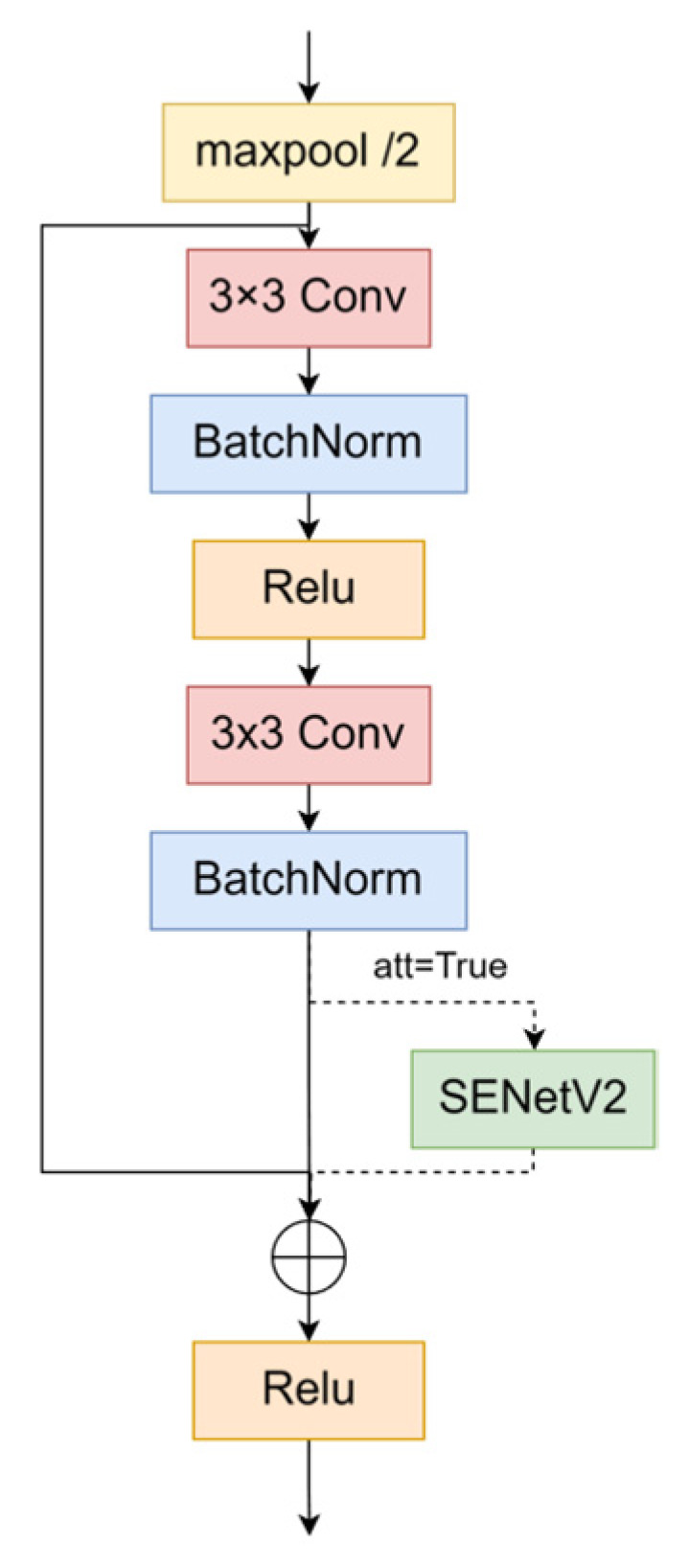
Structure of BasicBlock_SENetV2. The att parameter indicates whether the SENetV2 module is activated.

We reconstructed the BasicBlock module by incorporating the SENetV2 module to capture both the channel and global information more effectively. Its structure is shown in Figure 3.

**Figure 4:**
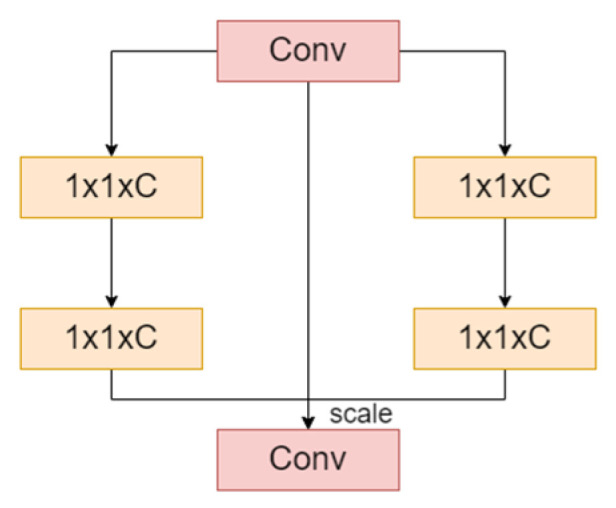
Structure of SENetV2. “1×1×C” represents 1×1 convolution layers with C channels. Conv layers perform convolution operations, and “scale” adjusts feature recalibration.

Traditional convolutional neural networks excel at extracting local features but struggle with capturing global information and the intricate relationships between channels. The SENetV2 module addresses these limitations by integrating squeezing and excitation operations, which enhances the network’s ability to acquire global features through a multibranch fully connected layer. The structure of SENetV2 is shown in Figure 4.

**Figure 5:**
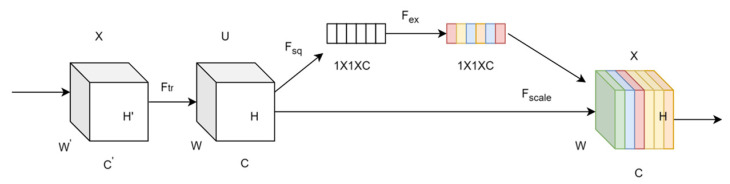
Structure of the SE module. The input tensor X undergoes transformation through squeezing ($F_{sq}$) and excitation ($F_{ex}$) operations, followed by a scaling ($F_{scale}$) step to recalibrate feature maps.

The aggregated layers from the squeezing operation are cascaded and passed through the FC layer. A scaling operation is then performed, multiplying the output by the module inputs to restore the original dimensions. The sequence of operations within a residual module is expressed as follows:

(5) SEnetV2=x+F(x⋅Ex(∑Sq(x)))

where x denotes input, F refers to the operations that modify the input, including batch normalization and dropout, Sq denotes the squeeze operation, and Ex denotes the excitation operation. The structure of the squeeze and excitation (SE) module, which enhances channel dependency, is shown in Figure 5.

First, the input feature map X is transformed to produce feature map U. The $F_{tr}$ can be formulated as follows:

(6) $$U_{C}=V_{C}*X=\sum_{S=1}^{C^{\prime }}V_{C}^{S}*X^{S}$$

where X∈R(H’×W’×C’) represents the input feature map, U∈R(H×W×C) denotes the output feature map, V indicates a set of learned filter kernels, $V_{C}\;$ represents the parameters of the filter, $V_{C}^{S}$ denotes a 2D spatial kernel, $X^{S}$ refers to the feature map corresponding to the convolutional window of size *S*, and ∗ represents a convolution operation.

$F_{sq}$ using global average pooling across channels, the feature map $U_{C\;}\;$ with dimensions H×W×C is directly compressed into an 1×1×C feature vector Z. This operation reduces the features of each channel to a single value, enabling the resulting channel data to capture contextual information and alleviate issues related to channel dependency. The formula used is as follows:

(7) $$Z_{C}=F_{sq}(U_{C})=\frac{1}{H\times W}\sum_{i=1}^{H}\sum_{j=1}^{W}u_{c}(i,j)$$

The vectors are processed through two fully connected layers. The first layer reduces dimensionality and applies a ReLU activation function for nonlinear transformation, while the second layer restores dimensionality and uses a sigmoid activation function to compute the channel weights. The original feature map is then multiplied by these learned channel weights, producing a calibrated feature map that emphasizes important features.

#### 3.2.2. CG Block

**Figure 6:**
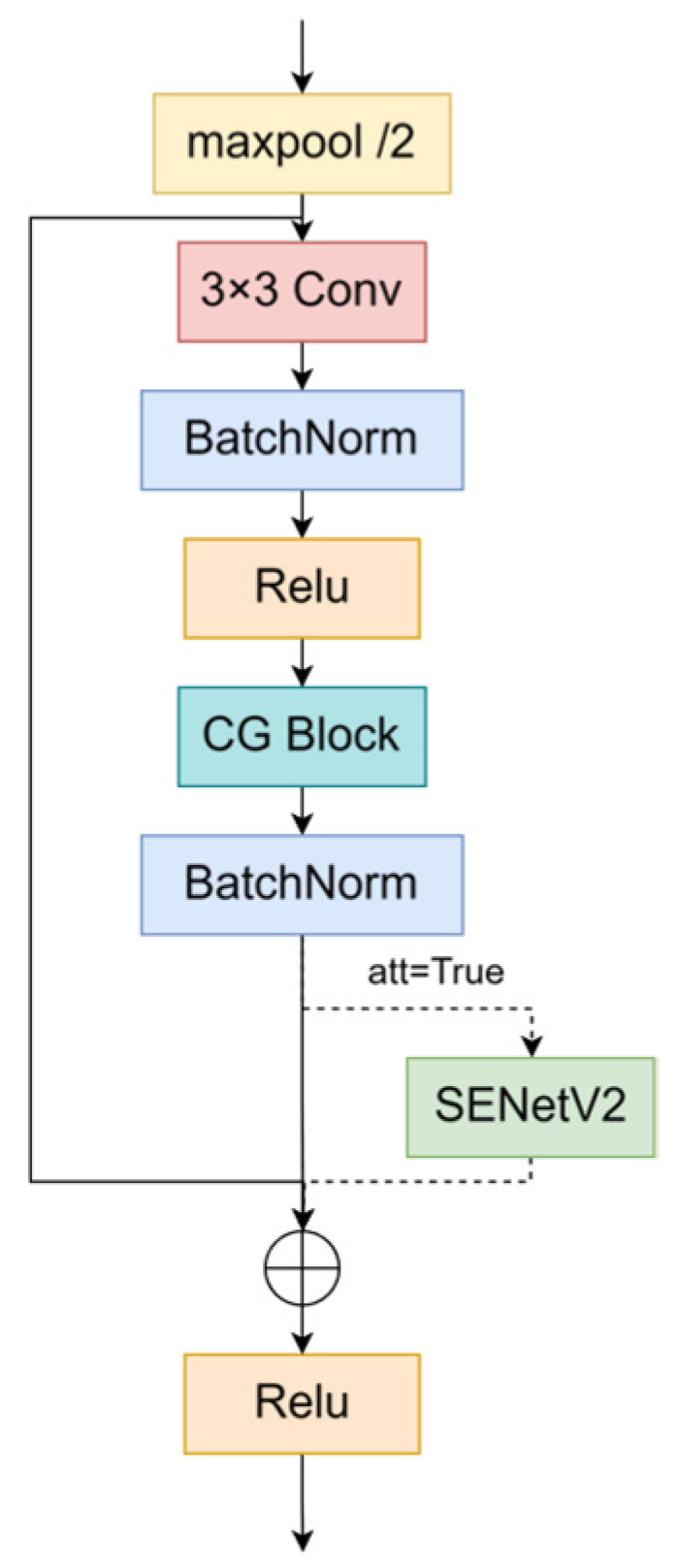
Structure of the BasicBlock_ContextGuided. The att parameter was used to train the SENetV2 module.

Vehicle logos often occupy only a small portion of an image, which results in sparse feature information. To address this and reduce the network’s parameters, the BasicBlock module is restructured by integrating the CG block from the lightweight and efficient semantic segmentation network CGNet. The structure is illustrated in Figure 6.

**Figure 7:**
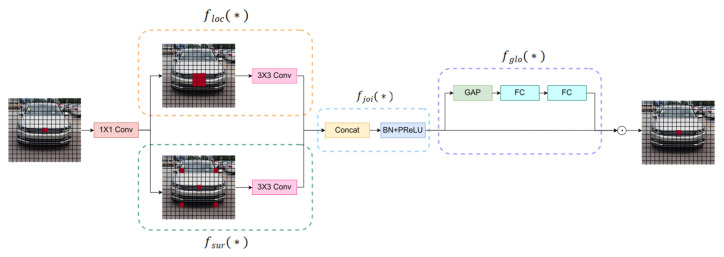
Structure of the GCBlock. Local feature extractor $f_{loc}(*)$, surrounding context extractor $f_{sur}(*)$, joint feature extractor ${\;f}_{joi}(*)$, and global context extractor $f_{glo}(*)$.

The CG block is inspired by the human visual system’s use of contextual information to interpret scenes, effectively processing and integrating features at different levels. By incorporating this lightweight semantic segmentation module into the object detection network, the efficiency of the network was enhanced. The structure of the CG block is shown in Figure 7.

The process begins with a 1 × 1 convolutional layer that generates the initial feature map. This feature map is then processed through $f_{loc}(*)$ and $f_{sur}(*)$, which focus on extracting the local and contextual features. To further increase computational efficiency, both components utilize depthwise separable convolution (DSC).

**Figure 8:**
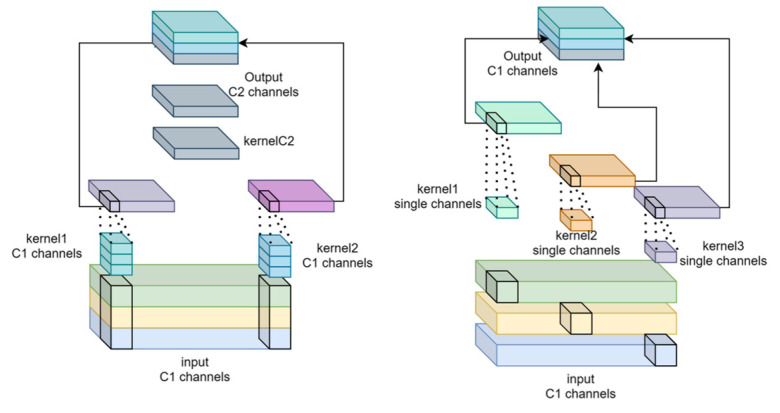
Calculation process of the SC and DSC. DSC uses separate kernels for each input channel to improve computational efficiency compared to SC.

As shown in Figure 8, standard convolution (SC) performs dense computations across all channels, whereas depthwise separable convolution (DSC) divides this process into two operations: depthwise convolution, which applies a filter to each input channel independently, and pointwise convolution, which combines the outputs from depthwise convolution using a 1 × 1 kernel. This separation significantly reduces the number of parameters and computational complexity, thereby lowering memory usage and minimizing the risk of overfitting without compromising model performance.

The $f_{loc}(*)$ component uses a 3 × 3 standard convolutional layer to capture the local features from the localized regions of the image. In parallel, $f_{loc}(*)$ uses dilated convolution, which introduces spacing within the convolution kernel, thereby expanding the receptive field without increasing the number of parameters or computational cost. This enables the model to capture richer contextual information, thereby enhancing its ability to recognize vehicle logos in complex scenes.

$f_{joi}(*)$ obtains joint features from the outputs of $f_{loc}(*)$ and $f_{sur}(*)$ and subsequently fuses these local and contextual features through layer connections, batch normalization, and parameterized rectified linear unit (PReLU) activation. The PReLU enhances the model’s expressive capability by introducing learnable parameters that enable adaptive activation for each neuron.

Finally, the global context is extracted from the joint features via $f_{glo}(*)$. This process involves capturing the global information of the input image by aggregating the features through a global average pooling layer. This global context was then refined via a multilayer perceptron (MLP). A scaling layer is applied to weigh the joint features, emphasize their importance, and further process the information through two FC layers to optimize the final feature representation.

### 3.3. Neck Improvements

**Figure 9:**
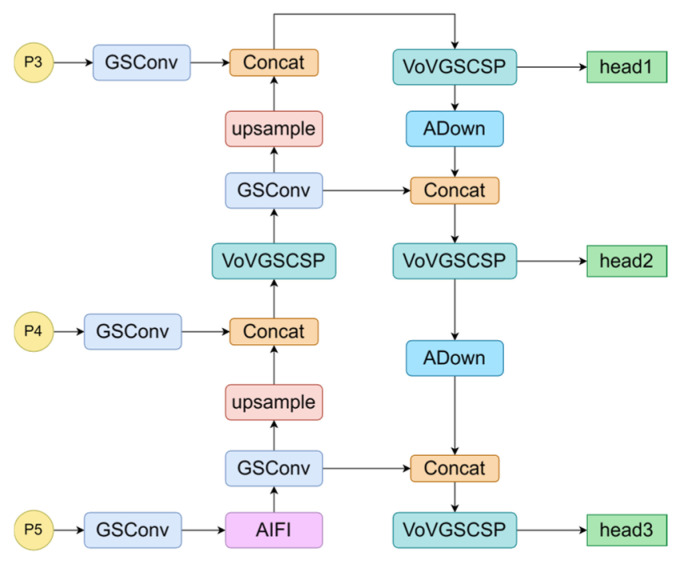
Structure of the Slim-Neck architecture.

RT-DETR uses only a single Transformer encoder layer in the neck network, which may result in the loss of semantic information. To mitigate this issue, the Slim-Neck architecture is employed to reduce the computational complexity and inference time while preserving semantic information. Additionally, a lightweight ADown module was introduced to replace the original downsampling convolution, further reducing the computational overhead. The model structure is shown in Figure 9.

#### 3.3.1. GSConv

In the backbone network, each module progressively reduces the spatial resolution while increasing the number of channels, capturing higher-level features, but causing some loss of semantic information. To balance semantic preservation with computational efficiency, the GSConv module was employed, ensuring faster inference without sacrificing semantic integrity, which is crucial for long-range vehicle logo detection.

**Figure 10:**
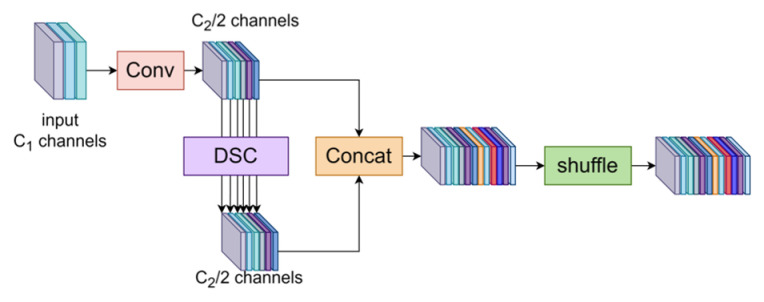
Structure of the GSConv. Conv refers to the SC.

As shown in Figure 10, SC improves implicit channel connections and preserves more feature information but increases computational complexity. In contrast, DSC reduces complexity by limiting these connections, potentially leading to information loss. The GSConv module effectively balances these aspects by maximizing channel connections to maintain semantic integrity while optimizing efficiency. Figure 9 illustrates the model structure, where C1 and C2 represent the input and output channel counts, respectively. Shuffling is employed to evenly distribute the features from the SC across those generated by the DSC, facilitating uniform feature exchange across channels without adding unnecessary complexity.

#### 3.3.2. VOVGSCSP

**Figure 11:**
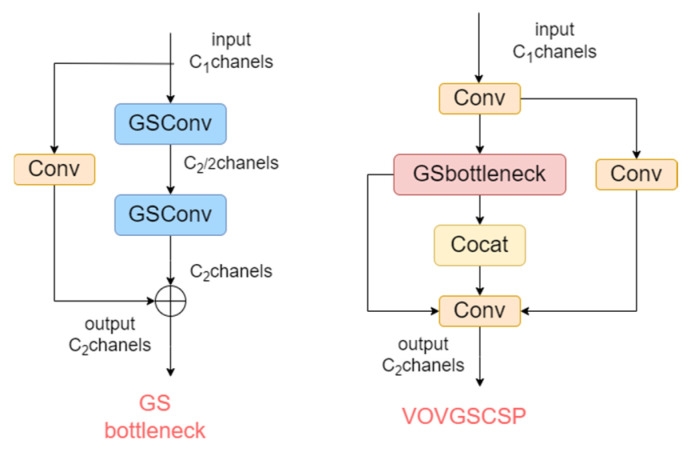
Structures of the GS bottleneck module and the VOVGSCSP module.

The GS bottleneck module built on GSConv improves the nonlinear representation of features and enhances information reuse. In addition, the VOVGSCSP module employs a one-shot aggregation strategy to design an efficient cross-stage partial network (CSP) module, VOVGSCSP. This approach minimizes the computational complexity and inference time while maintaining accuracy. The structure is shown in Figure 11.

#### 3.3.3. ADown

**Figure 12:**
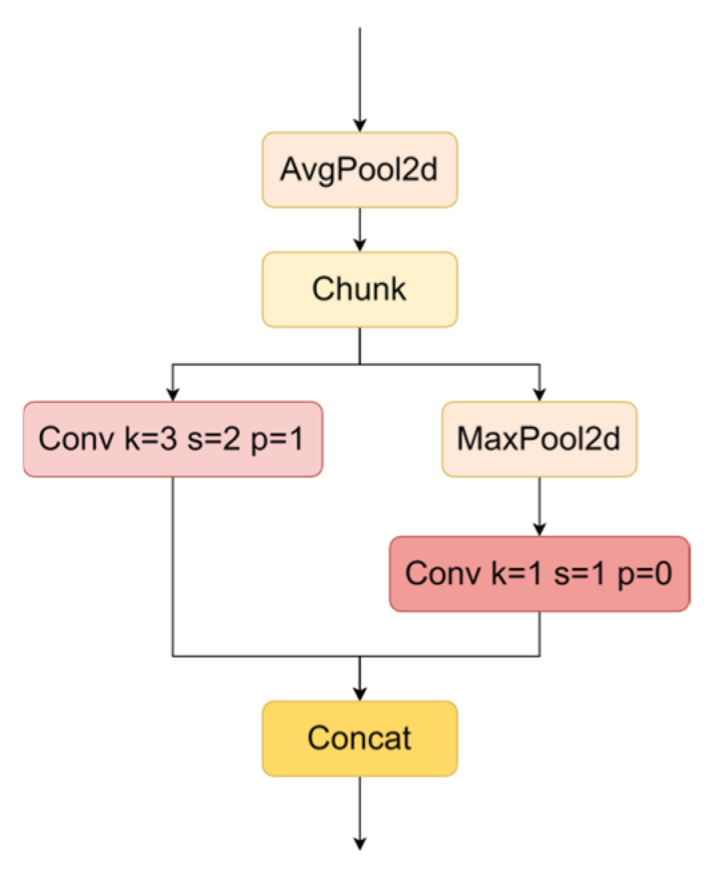
Structure of the Adown; k represents the kernel size, s is the stride, and p is the padding.

To optimize the downsampling operation, the ADown method is integrated into the GSConv module, as illustrated in Figure 12. The ADown module begins with average pooling of the input feature maps, reducing the spatial dimensions while preserving essential feature information. The pooled output is then concatenated with the original feature map and processed through a subsequent chunk operation that splits it into two parallel branches.

In the first branch, a 3 × 3 convolution is used for both downsampling and feature extraction, while the second branch employs max pooling followed by a 1 × 1 convolution. By processing these branches simultaneously, the ADown module captures and integrates features from multiple perspectives. The outputs of these branches are concatenated along the channel dimension to produce the final downsampling result. This approach maintains feature integrity while improving computational efficiency and downsampling performance through parallel processing and feature fusion.

## 4. Experimental Design and Interpretation of Results

### 4.1. Experimental Equipment and Evaluation Indicators

The experiments were conducted on a system running Windows 11, equipped with a 13th Gen Intel^®^ Core^TM^ i9-13900K 3.00 GHz CPU, 128 GB RAM (Intel Corporation, Santa Clara, CA, USA), and an NVIDIA RTX A6000 GPU (Nvidia, Santa Clara, CA, USA). The deep learning framework used PyTorch 2.0.1 and CUDA 11.7.

The IoU threshold is set between 0.5 and 0.95 to distinguish between foreground and background object detection. The evaluation metrics employed include average precision (AP), recall, and mean average precision (mAP). AP is the mean precision over the IoU range of 0.5 to 0.95, whereas recall is assessed from 0 to 1. In practice, AP is approximated by calculating the area under the precision-recall curve using a finite sum over discrete recall values. This provides a comprehensive measure of the model’s performance across different thresholds, with AP representing the mean of precision values at different IoU levels. The formula used is as follows:

(8) $$\mathrm{AP}=\int_{0}^{1}\mathrm{Precision}(r)dr$$

mAP is the mean value of the average precision AP for all categories. The formula for calculating the average accuracy across n classes is as follows:

(9) $$\mathrm{mAP}=\frac{1}{N}\sum_{i=1}^{N}{\mathrm{AP}}_{i}$$

### 4.2. Dataset

**Figure 13:**
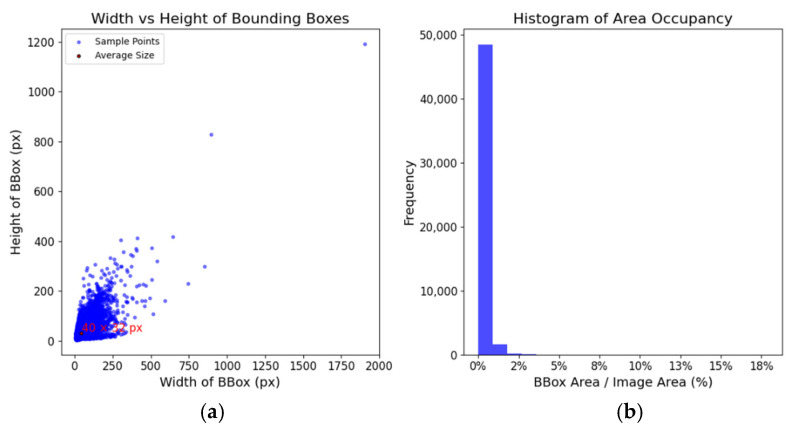
(**a**) Width vs. height of bounding boxes; (**b**) histogram of BBox area occupancy.

As shown in Figure 13, the VLD-45 dataset had an average object size of 40 × 32 pixels, with many objects exceeding 200 × 200 pixels, predominantly occupying less than 2% of the image area. The COCO dataset defines objects smaller than 32 × 32 pixels as small in absolute terms, whereas those occupying ≤2% of the image area are classified as small in relative terms. Therefore, the VLD-45 dataset can only be considered small under a relative definition, which limits its effectiveness in real-world scenarios. To address the challenges of long-range detection, the VLD-Micro dataset was specifically created.

**Figure 14:**
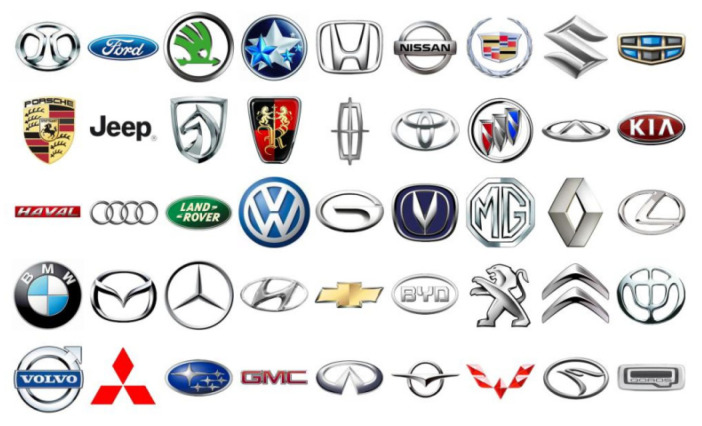
Vehicle labeling dataset object categories.

First, images of vehicle logos from the VLD-45 dataset that depicted partial views of vehicles that were not representative of real-world scenes or with object sizes larger than 40 × 32 pixels were filtered out, totaling 11,969 images. The remaining images were obtained through web scraping from Baidu, resulting in microscale vehicle logo images with object sizes smaller than 24 × 24 pixels. This process draws on the structure and principles of the Pascal VOC dataset and includes manual annotations. Following the annotation guidelines, 50,289 vehicle logos were manually annotated in the dataset. This dataset comprises 45 categories, as shown in Figure 14, with a total of 45,000 images. The dataset was divided into training, validation, and test sets in a ratio of 5:3:2, with 1000 images in each category: 445 images allocated to the training set, 333 allocated to the validation set, and 222 allocated to the test set.

### 4.3. Comparison

The performance of the improved backbone network was evaluated against several well-known lightweight backbone networks, including CNN backbones (MobileNetv3, EfficientNetv2, ShuffleNetv2, and VanillaNet13) and ViT models (RepViT and EfficientViT). This comparison, detailed in Table 2, involves substituting the RT-DETR backbone with these alternatives. The improved network, featuring 18.74 million parameters, is comparable to MobileNetv3 and ShuffleNetv2 in terms of size. However, with a computational cost of 55.4 GFLOPS, it has the lowest FLOPS among all the models, indicating superior computational efficiency. The mAP@50:95 also surpasses that of mainstream backbone networks. In terms of real-time performance, the network achieves 68.11 FPS, significantly outperforming other models, including a notable 14.38 FPS advantage over EfficientViT. Overall, the improved network excels in computational efficiency, detection accuracy, and real-time performance, demonstrating exceptional capability for vehicle logo detection.

To assess the detection accuracy and efficiency of the improved algorithm, we compared the enhanced RT-DETR with several well-established models, including both single-stage and two-stage algorithms, as well as DETR, using the VLD-Micro dataset. YOLOv8 and YOLOv9 were selected to represent the single-stage algorithms. YOLOv8 utilized pretrained weights from YOLOv8n, while YOLOv9 employed weights from YOLOv9-T and was trained using a dual-backbone network. For the two-stage algorithm, Faster R-CNN was chosen for its accuracy, with ResNet50 as the backbone. RT-DETR was included as a representative of the DETR family to evaluate the performance of Transformer-based models.

The evaluation of the improved model involves several key metrics: mAP@50:95 for detection accuracy, inference time (Times), and both Params and FLOPS for model complexity. As shown in Table 3, while YOLOv8 has a faster inference time of 6.8 ms, our model processes at 8.2 ms, which is still within the real-time performance range. However, our model achieved a 1.6% higher mAP@50-95. Additionally, our model’s parameter count is only 37% of YOLOv9’s, with just a 0.4% reduction in accuracy, and Faster R-CNN has 2.2 times more parameters with 7.4% lower accuracy.

While YOLOv8 is more efficient in terms of speed and FLOPS, our model achieved a competitive 55.4G FLOPS, representing a 36.7% reduction compared to the original RT-DETR. Despite YOLOv8’s lightweight design, our model offers superior accuracy and a better balance between computational efficiency and performance, making it especially effective for detecting distant or small objects, such as vehicle logos.

In conclusion, the improved model strikes an optimal balance between accuracy, speed, and efficiency, making it well suited for real-time applications that require precise detection of small or distant objects like vehicle logos.

### 4.4. Comparison of Test Results

**Figure 15:**
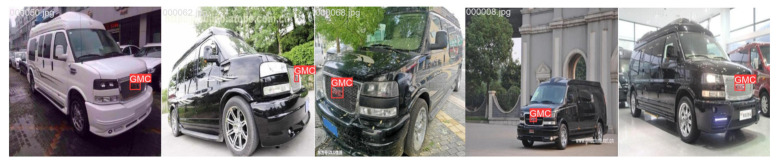
Ground truth.

**Figure 16:**
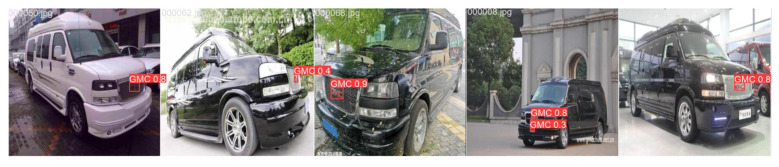
Results of RT-DETR.

**Figure 17:**
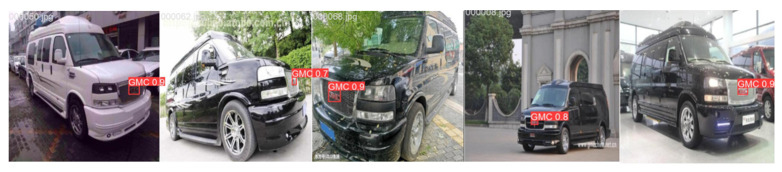
Results of our method.

The visual results of vehicle logo detection across different scenarios, comparing the improved model with RT-DETR, are shown in Figure 15, Figure 16 and Figure 17. Figure 15 shows the ground truth annotations for the original images, while Figure 16 and Figure 17 show that the improved model achieves a slightly higher accuracy than RT-DETR does. These results show that the improved model not only improves detection accuracy but also enhances adaptability and robustness in various scenes. This advancement enabled the model to handle vehicle logo detection more effectively in complex environments for practical applications.

### 4.5. Grad-CAM Visualization

**Figure 18:**
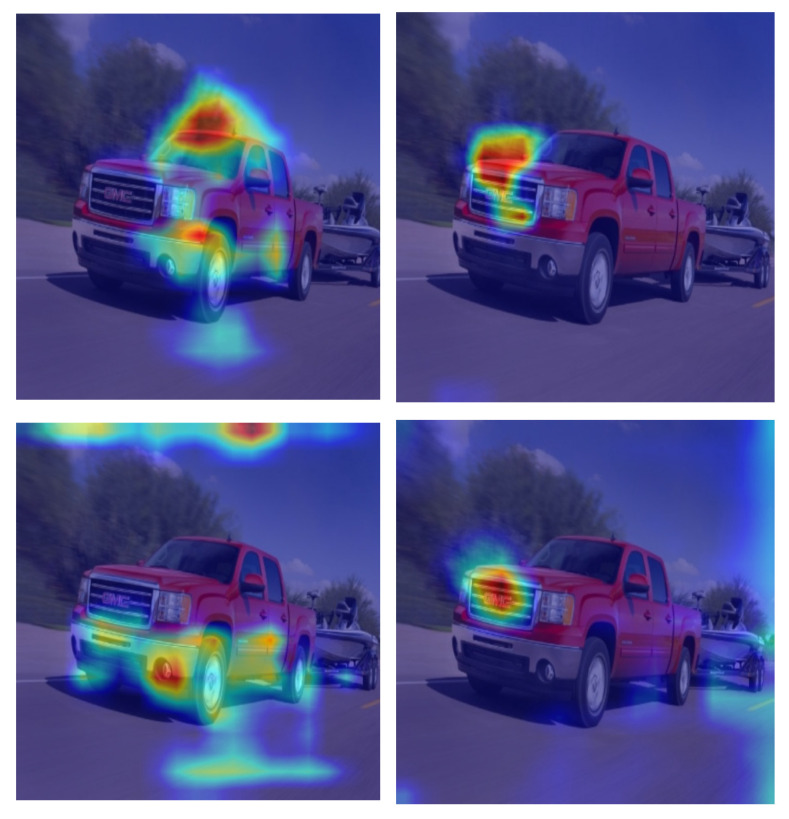
Grad-CAM visualization results for the RT-DETR and improved models, with warmer colors showing higher attention and cooler colors indicating lower attention.

To further demonstrate the effectiveness of the proposed method, we employed the Grad-CAM technique for visualization and analysis, as shown in Figure 18. In the figure, the annotated regions correspond to the predicted vehicle logo locations, showing how Grad-CAM highlights the relevant features. For each pair of images, the left image shows the Grad-CAM visualization results for RT-DETR, whereas the right image shows the results for the improved model. These visualizations illustrate how the model focuses on different features after processing through backbone and neck networks. A comparison of the two sets of images reveals that the improved RT-DETR model focuses more accurately on vehicle logo regions and captures critical details more efficiently during feature extraction.

### 4.6. Ablation Experiment

To demonstrate the impact of the proposed improvements, we conducted ablation experiments, as listed in Table 4. The experiments were performed using the VLD-Micro dataset, with each configuration trained for 72 epochs. To ensure consistency, we used the same hyperparameters across all experiments: a learning rate of 0.0001, a batch size of 16, and an input image size of 640 × 640 pixels. The results indicate that the Slim-Neck architecture slightly reduces the number of model parameters, while providing a modest improvement in accuracy. SENetv2, on the other hand, enhances detection accuracy without increasing the model’s parameter count. CGBlock effectively reduced the number of model parameters while maintaining accuracy.

## 5. Conclusions

In this study, we introduce a lightweight algorithm based on RT-DETR, specifically designed to enhance long-range vehicle logo detection. This approach integrates a ResNet-34 backbone network augmented with SENetV2 and CGBlock to improve shallow features and global information, thereby improving the retention of small-object features. The neck network uses the Slim-Neck architecture coupled with the ADown module to refine the downsampling process. Furthermore, we developed the VLD-Micro dataset, which includes vehicle logos of significantly smaller sizes than those found in existing datasets, replicating conditions where logos can appear as small as 24 × 19 pixels or less, thus simulating real-world long-distance detection tasks. Experiments on the VLD-Micro dataset achieved an mAP@50:95 of 0.698, indicating a 1.5% improvement over the baseline RT-DETR model, with an inference speed of 8.2 ms and a 36.7% reduction in FLOPS. These results underscore the model’s ability to accelerate inference and lower computational costs while maintaining high accuracy, making it well suited for deployment in resource-constrained devices.

However, despite these advancements, the proposed model faces challenges in highly variable environments such as extreme lighting conditions and occlusions. Future work will focus on enhancing the robustness of the model by integrating advanced data augmentation techniques and multiscale feature integration. This initiative aims to further improve the proposed algorithm and its methodologies to better satisfy real-world requirements.

## Figures and Tables

**Table 1:** ResNet comparison experiment results.

Backbone	Params (M)	FLPOS (G)	mAP@50	mAP@50:95	FPS
RT-DETR-L	32.79	105.1	0.967	0.687	26.02
ResNet-18	**19.94**	**57.4**	0.967	0.686	50.26
ResNet-34	30.05	87.6	0.966	0.688	**66.11**
ResNet-50	42.90	134.6	**0.970**	**0.691**	27.37

**Table 2:** Performance comparison of different lightweight backbone networks.

Backbone	Params(M)	FLPOS(G)	Map@50	mAP@50:95	FPS
MobileNetv3	18.54	57.2	0.953	0.663	34.18
EfficientNetv2	28.47	70.1	0.967	0.692	43.91
ShuffleNetv2	18.80	59.5	0.92	0.626	13.7
RepViT	13.39	37.0	0.97	0.691	15.3
EfficientViT	**10.76**	**27.4**	0.947	0.66	53.73
VanillanNet13	26.29	81.0	0.921	0.631	11.7
Our Method	18.74	55.4	**0.976**	**0.698**	**68.11**

**Table 3:** Performance comparison of different networks.

Category	YOLOv8	YOLOv9	Faster R-CNN	RT-DETR	Our Method
GMC	0.676	**0.699**	0.651	0.679	0.687
Audi	**0.75**	0.743	0.677	0.72	0.732
BAOJUN	0.708	0.724	0.696	0.722	**0.741**
HONDA	0.698	0.726	0.670	0.712	**0.728**
PEUGEOT	0.643	**0.689**	0.604	0.667	0.672
Buick	0.743	**0.749**	0.676	0.712	0.741
Soueast Motor	0.701	0.702	0.676	0.72	**0.759**
QOROS	0.685	**0.705**	0.648	0.695	0.694
Trumpchi	0.633	0.67	0.625	0.687	**0.692**
Chevrolet	**0.709**	0.68	0.622	0.66	0.698
Citroen	**0.706**	0.706	0.632	0.672	0.682
Infiniti	0.602	**0.721**	0.647	0.694	0.707
SKODA	0.606	0.605	0.610	0.625	**0.631**
Porsche	**0.691**	0.601	0.574	0.61	0.618
HYUNDAI	0.685	0.683	0.652	0.675	**0.689**
Huachen Auto Group	0.661	0.681	0.617	0.67	**0.678**
Volvo	0.658	0.742	0.689	0.74	**0.748**
Mitsubishi	**0.672**	0.67	0.583	0.641	0.653
Subaru	**0.696**	0.678	0.635	0.661	0.687
SGMW	**0.713**	0.707	0.669	0.69	0.708
BAIC GROUP	0.678	0.744	0.686	0.741	**0.745**
Venucia	**0.751**	0.691	0.648	0.694	0.697
Cadillac	0.663	**0.759**	0.695	0.737	**0.759**
GEELY	0.688	0.68	0.630	**0.692**	0.689
SUZUKI	0.545	0.71	0.668	0.707	**0.716**
Jeep	**0.743**	0.593	0.519	0.568	0.596
ROEWE	0.521	0.718	0.692	0.73	**0.738**
LINCOLN	**0.708**	0.556	0.476	0.525	0.521
TOYOTA	0.728	0.717	0.656	0.697	**0.765**
NISSAN	0.689	**0.761**	0.723	0.741	0.752
KIA	0.605	0.692	0.658	0.699	**0.7**
Chery	0.588	**0.643**	0.566	0.622	0.618
HAVAL	**0.675**	0.627	0.556	0.61	0.612
Renault	**0.737**	0.696	0.615	0.676	0.677
LEXUS	0.699	**0.756**	0.714	0.753	0.757
Ford	0.653	0.726	0.711	0.722	**0.732**
BMW	**0.765**	0.683	0.626	0.664	0.668
MAZDA	**0.782**	0.776	0.721	0.748	0.762
Mercedes-Benz	0.699	**0.779**	0.706	0.749	0.761
BYD	0.702	0.718	0.682	0.707	**0.732**
FAW Haima	0.747	**0.78**	0.675	0.735	0.744
LAND ROVER	0.648	0.703	0.657	0.698	**0.706**
Volkswagen	0.693	**0.719**	0.647	0.692	0.696
CHANGAN	0.671	0.67	0.601	0.657	**0.661**
Morris Garages	0.744	**0.746**	0.682	0.731	0.732
**mAP@50-95**	0.687	**0.701**	0.646	0.688	0.698
**Times (ms)**	**6.8**	57.8	181.7	15.1	8.2
**Params (M)**	**3.02**	50.8	41.58	30.05	18.74
**FLOPS (G)**	**8.2**	237.2	134.45	87.6	55.4

**Table 4:** Results of ablation experiments.

Model	Params (M)	FLOPS (G)	mAP@50	mAP@50:95
RT-DETR	30.05	87.6	0.966	0.688
RT-DETR + SlimNeck	29.21	83.6	0.968	0.690
RT-DETR + SlimNeck + SENetv2	29.85	83.6	0.976	0.698
RT-DETR + SlimNeck + SENetv2 + CGBlock	**18.74**	**55.5**	**0.976**	**0.698**

## Data Availability

The original contributions presented in the study are included in the article and further inquiries can be directed to the corresponding author.
